# The COVID-19 response illustrates that traditional academic reward structures and metrics do not reflect crucial contributions to modern science

**DOI:** 10.1371/journal.pbio.3000913

**Published:** 2020-10-16

**Authors:** Adam J. Kucharski, Sebastian Funk, Rosalind M. Eggo

**Affiliations:** Centre for Mathematical Modelling of Infectious Diseases, London School of Hygiene & Tropical Medicine, London, United Kingdom

## Abstract

The COVID-19 pandemic has motivated many open and collaborative analytical research projects with real-world impact. However, despite their value, such activities are generally overlooked by traditional academic metrics. Science is ultimately improved by analytical work, whether ensuring reproducible and well-documented code to accompany papers, developing and maintaining flexible tools, sharing and curating data, or disseminating analysis to wider audiences. To increase the impact and sustainability of modern science, it will be crucial to ensure these analytical activities—and the people who do them—are valued in academia.

The emergence of COVID-19 has highlighted the importance of analytical research. In the first six months of the pandemic, researchers from around the world collated and curated valuable open data sources [1,2], as well as living reviews of key epidemiological parameters [3]. Real-time development of statistical and modelling pipelines has enabled ongoing situational awareness, such as tracking of the reproduction number [4,5]. Rapid reports have provided crucial early analysis of virus evolution, transmission and severity [6–8], and interactive online apps have turned static results into flexible tools [9,10]. Alongside this analysis, open source models and data processing packages have enabled wider applications of new methodology [11,12]. These initiatives have been instrumental in supporting worldwide responses to the pandemic.

Such examples share several common features; they are open, collaborative, generate novel insights, and have immediate real-world impact. In many ways, they reflect the best aspects of scientific progress. But there is another feature they have in common: despite the value of these projects, they are outputs that are generally not captured by traditional academic metrics.

Two central activities mark the academic career path: obtaining research grants and publishing journal articles. These are in turn viewed through summary metrics, such as journal brand as a measure of quality and position in an author list as a measure of research contribution. These metrics may have originated with good intentions, namely to motivate dissemination of high quality science and ensure that people can be recognised for their work. But as with other simple performance metrics, measurements that were designed to track activity have instead become measurements that shape it. Because science has historically not been set-up to motivate rapid, open data sharing, there have been delays in the release of crucial data during outbreaks [13]. With publication count highly valued, analysis may also be delayed as teams ‘salami slice’ the underlying results into multiple papers. And because researchers have not been given the capacity, time or incentives to make their computer code open and accessible, much code is either unpublished or unusable.

Against a background of limited time and short-term contracts, researchers have had to decide whether to prioritise the measurable outputs that are beneficial—or even essential—to their own advancement and career survival, or whether to spend time on the types of analytical work described above. These analytical activities undoubtedly improve the robustness and reach of science, but without incentivisation or enforcement, they become voluntary. Writing reproducible and well documented code to accompany papers, sharing and curating data, or disseminating work to wider audiences are optional add-ons. Even if researchers believe these activities to be important, and commit time to them, the more extensive versions of this work—such as maintaining flexible, reusable code and packages—will substantially reduce the number of papers they can produce. As a result, there will be limited opportunities for career advancement among analytical specialists employed in traditionally academic roles or academic researchers who choose to focus on analytical work. Although some academics eventually develop reputations for their non-traditional activities, these projects will have often been ‘subsidised’ by a career platform built on traditional, measurable outputs.

The choices can be even more stark when researchers work on policy-related analysis involving sensitive, unpublishable data. During the COVID-19 pandemic, many researchers, across many countries, have put research projects and papers on hold to contribute to analytical policy work that is difficult to quantify in traditional metrics. Much of this work consists of ongoing situational awareness or scenario analysis, rather than discrete publishable outputs. As a result, these activities often fall outside the remit of the university-wide assessments such as the UK Research Excellence Framework, which typically ties a specific study to a specific impact.

Some might argue that scientists who dedicate time to activities outside traditional metrics are misinterpreting their role. Developing a tool, such as writing an R package or creating a dashboard, perhaps does not fit into the traditional interpretation of scientific work, which is focused on generation of discrete pieces of knowledge. But creating and maintaining projects that can provide insights both directly (through ongoing analysis and dissemination) and indirectly (through later reuse by others) ultimately improves science.

In the long-term, support for analytical work reduces inefficiency caused by repetition—such as multiple teams compiling the same dataset or redeveloping the same tools—and the risk of errors from individuals developing code in isolation. In order to support these activities, funders and employers will need to look beyond traditional metrics when assessing the performance of scientific teams. For example, instead of asking applicants for grants, tenure or employment simply for a list of publications, a broader portfolio should be permissible that includes software, data sets and analytical tools, even where these are not published in the format of pre-prints or peer-reviewed papers. The funding landscape also needs to reflect the scientific need for research involving development of analytical tools, as well as their accompanying maintenance and engagement with the community of users. Such efforts could complement wider initiatives to improve the integrity of research through open science, such as the Hong Kong Principles, which encourage recognition of the value of non-traditional research outputs, including replication efforts and open methods and data [14].

While some technical fields have started embracing research software engineering roles [15], there are limited opportunities in fields such as the biomedical sciences. As demand for analytical work and software engineering increases in biology and beyond, it will become impossible to recruit and retain people with this expertise if there is no clear career path for them. Change will require leadership at all levels—from funders to senior academics—to encourage activities that have previously been neglected, even if this means re-evaluating the balance of their own historical incentives and outputs.

The COVID-19 pandemic has shown that many researchers are highly motivated to produce work that has immediate impact, even if it is beyond the scope of traditional academic metrics. Now is the time to change the incentive structure to recognise these efforts. By ensuring crucial analytical activities—and the people who do them—are valued in academia, we can enable a more collaborative, impactful and sustainable future for science.
